# Multifunctional Amphiphilic Biocidal Copolymers Based on N-(3-(Dimethylamino)propyl)methacrylamide Exhibiting pH-, Thermo-, and CO_2_-Sensitivity

**DOI:** 10.3390/polym17141896

**Published:** 2025-07-09

**Authors:** Maria Filomeni Koutsougera, Spyridoula Adamopoulou, Denisa Druvari, Alexios Vlamis-Gardikas, Zacharoula Iatridi, Georgios Bokias

**Affiliations:** 1 Department of Chemistry, University of Patras, GR-26504 Patras, Greece; marifili.koutsougera@gmail.com (M.F.K.); up1064176@upnet.gr (S.A.); druvari@upatras.gr (D.D.); avlamis@upatras.gr (A.V.-G.); bokias@upatras.gr (G.B.); 2 Department of Materials Science, University of Patras, GR-26504 Patras, Greece; 3 Foundation for Research and Technology-Hellas, Institute of Chemical Engineering Sciences (FORTH/ICE-HT), Stadiou Street, GR-26504 Patras, Greece

**Keywords:** N-(3-(dimethylamino)propyl)methacrylamide, methyl methacrylate, amphiphilic polymers, self-assembly, pH-responsive, thermo-responsive, CO_2_-sensing, antibacterial

## Abstract

Because of their potential “smart” applications, multifunctional stimuli-responsive polymers are gaining increasing scientific interest. The present work explores the possibility of developing such materials based on the hydrolytically stable N-3-dimethylamino propyl methacrylamide), DMAPMA. To this end, the properties in aqueous solution of the homopolymer PDMAPMA and copolymers P(DMAPMA-co-MMA_x_) of DMAPMA with the hydrophobic monomer methyl methacrylate, MMA, were explored. Two copolymers were prepared with a molar content x = 20% and 35%, as determined by Proton Nuclear Magnetic Resonance (^1^H NMR). Turbidimetry studies revealed that, in contrast to the homopolymer exhibiting a lower critical solution temperature (LCST) behavior only at pH 14 in the absence of salt, the LCST of the copolymers covers a wider pH range (pH > 8.5) and can be tuned within the whole temperature range studied (from room temperature up to ~70 °C) through the use of salt. The copolymers self-assemble in water above a critical aggregation Concentration (CAC), as determined by Nile Red probing, and form nanostructures with a size of ~15 nm (for P(DMAPMA-co-MMA_35_)), as revealed by transmission electron microscopy (TEM) and dynamic light scattering (DLS). The combination of turbidimetry with ^1^H NMR and automatic total organic carbon/total nitrogen (TOC/TN) results revealed the potential of the copolymers as visual CO_2_ sensors. Finally, the alkylation of the copolymers with dodecyl groups lead to cationic amphiphilic materials with an order of magnitude lower CAC (as compared to the unmodified precursor), effectively stabilized in water as larger aggregates (~200 nm) over a wide temperature range, due to their increased ζ potential (+15 mV). Such alkylated products show promising biocidal properties against microorganisms such as *Escherichia coli* and *Staphylococcus aureus*.

## 1. Introduction

Stimuli-responsive polymers, namely polymers displaying abrupt reversible changes as a response to external stimuli (such as pH, temperature, light, magnetic fields, CO_2_, etc.) [1,2,3,4,5,6], represent an appealing class of “smart’’ materials, extensively explored the last decades for a variety of applications, including sensing, drug delivery, biomedical applications, etc. [7,8,9,10,11]. As compared to most common and more studied single- or dual-stimuli-responsive polymers, the design of multi-stimuli-responsive polymers offers the possibility for the preparation of elaborate intelligent materials, through the simultaneous control of several external parameters, presenting rich behaviors with multiple functions [12,13,14,15].

Poly(N-isopropylacrylamide) is certainly the most extensively studied water-soluble thermo-responsive polymer [16,17] presenting a Lower Critical Solution Temperature (LCST) behavior in water, expressed as a phase separation/turbidity upon heating above ~32 °C [18]. Starting from this paradigm, several classes of LCST polymers have been developed, the most common based on polymers of alkyl (meth)acrylamides, ethylene glycol, or N-alkyl-substituted aminoethyl (meth)acrylates [19,20,21,22]. The last class, i.e., weak polybases, has garnered considerable attention, since they can combine thermo-responsiveness with pH-responsiveness, through a careful selection of N-alkyl substituents for a delicate adjustment of the hydrophilic/hydrophobic balance. For instance, homopolymer poly(N,N-diethylaminoethyl methacrylate), PDEAEMA, is water-soluble only when protonated [23], whereas the homopolymer poly(N,N-dimethylaminoethyl methacrylate), PDMAEMA, is a pH-controlled LCST-type polymer presenting a cloud point temperature, Tcp, of 40–50 °C when unporotonated [24]. Such materials often offer a nice opportunity to develop CO_2_ sensors [25,26,27], since they turn to the charged protonated form, losing thus the LCST behavior, upon reacting with CO_2_ in water. A further advantage of these weak polybases is that they can undergo alkylation, giving rise to polymeric quaternary ammonium compounds, useful for gene delivery or biocidal applications, among other bioapplications [28,29,30].

A drawback of DMAEMA and DEAEMA is that the ester bond is susceptible to hydrolysis, giving hydrolysis products such as methacrylic acid, which may negatively affect the desired characteristics and behavior [31]. Replacing this ester bond by an amide bond in the case of the respective (meth)acrylamides could be the key solution to develop similar functional multi-responsive polymers, preventing hydrolysis. For example, the monomer N-2-diethylamino ethyl acrylamide, PDEAEAM, was synthesized and polymerized, leading to the respective thermo-, pH-, and CO_2_-responsive homopolymer [32]. Moreover, the incorporation of the CO_2_-switchable DEAEAM monomer in N-isopropylacrylamide-based polymers enabled the easy fine tuning of the polymer properties [33].

In contrast to DEAEAM, N-3-dimethylamino propyl methacrylamide, DMAPMA, is a commercially available and affordable monomer. Thus, polymers based on DMAPMA (either alkylated or not) have been proposed for applications in solution or as hydrogels such as functional adsorbents [34,35] and superadsorbents [36], shear thickeners [37], hybrid hydrogels with exceptional mechanical properties [38], perovskite layers for solar cells [39], pH- and temperature-driven actuators [40], injectable hydrogels [41], controlled drug release [42,43], and gene delivery [44]. Moreover, just like the (meth)acrylate counterparts, the tertiary amine group of DMAPMA can react with CO_2_ in the presence of water [45]. Thus, DMAPMA-based materials have been recently proposed for CO_2_-sensitive applications, such as pH-/CO_2_-switchable viscoelastic fluids [46], CO_2_-responsive sealants for cement applications [47], CO_2_ adsorbents [48,49,50,51], and sensors [52,53,54]. Finally, recent studies have focused on the development of DMAPMA-based antibacterial polymers [55,56] and surfaces [57].

A consequence of the short methyl substituents, as compared to the ethyl substituents in DEAEAM, is that the homopolymer poly(N-3-dimethylamino propyl methacrylamide), PDMAPMA, is less hydrophobic and does not present any LCST behavior, unless under strongly alkaline conditions. In fact, a cloud point of PDMAPMA of ~35 °C has been reported only when pH = 14 [58]. For this reason, when temperature-sensitive properties are desirable, the copolymerization of DMAPMA with thermosensitive (usually NIPAM) or hydrophobic monomers like styrene is often performed to achieve a subtle shift in the hydrophilic/hydrophobic balance towards hydrophobicity [40,59].

The main motivation of the present work was to further expand the class of thermo-responsive polymers through the adequate design of DMAPMA-based polymers. As compared to the main counterpart, namely DMAEMA-based copolymers, this novel family offers novel design opportunities and the advantages of the hydrolytically stable and relatively low cost DMPAPMA monomer. To this end, we first explicitly explored the LCST behavior of PDMAPMA as a function of pH and ionic strength. More important, as a consequence of the highly hydrophilic character of PDMAPMA (as compared to PDMAEMA), the efficient control of the LCST behavior over the whole useful temperature range of liquid water may be achieved through the copolymerization of DMAPMA with a hydrophobic monomer, for instance methyl methacrylate (MMA). Although these copolymers were derived by free radical copolymerization, they are amphiphilic materials and their chemical composition would possibly allow self-assembly in aqueous solution, as has been reported for similar systems [60,61]. The multi-responsiveness of these polymers in water was assessed in this work by examining the optical density changes with temperature (turbidimetry) under diverse pH conditions. CO_2_ sensitivity was explored through turbidimetry, ^1^H-NMR, and automatic total organic carbon/total nitrogen (TOC/TN) measurements. In addition, the self-assembly behavior of these materials was investigated by photoluminescent probing spectroscopy and dynamic light scattering.

Studies by our group have focused on the design of alkylated polymers and the evaluation of their properties, including their antimicrobial efficiency [37,62]. Along these lines, the alkylation of the aforementioned polymers with propyl-, dodecyl-, and hexadecyl-derivatives was conducted in order to explore their physicochemical properties as compared to the unmodified precursors and to assess their potential as antimicrobial materials.

## 2. Materials and Methods

### 2.1. Materials

N-(3-(dimethylamino)propyl)methacrylamide is a product of Alfa Aesar (Kandel, Germany) and was used as received. Methyl methacrylate (MMA), 2,2-azo-di-isobutyronitrile (AIBN), tetrahydrofuran (THF), the deuterated solvents dimethyl sulfoxide (DMSO-d_6_), deuterated water (D_2_O), potassium dihydrogen phosphate (KH_2_PO_4_), and Nile Red are products of Sigma-Aldrich (Steinheim, Germany). Sodium acetate reagent (CH_3_COONa) and the organic solvents hexane and petroleum ether were purchased from Carlo Erba (Barcelona, Spain). Sodium hydroxide (NaOH), sodium chloride (NaCl), and hydrochloric acid (HCl) were purchased from Merck (Merck KGaA, Darmstadt, Germany). Ultrapure water was prepared using an Arium Mini Water Purification System (Sartorius, Göttingen, Germany).

### 2.2. Synthesis of PDMAPMA Homopolymer

In a three-necked 100 mL spherical flask, equipped with a magnetic stirrer, 12 mL (66 mmol) of the DMAPMA monomer was dissolved in 60 mL THF. The solution was left for ~30 min under stirring conditions and degassed through nitrogen bubbling. Then, 54 mg (0.33 mmol, 0.5 mol% over the total monomer concentration) of the initiator AIBN was added and the system was stirred at 70 °C under a nitrogen atmosphere for 1 day. Afterwards, the reaction mixture was precipitated in excess volumes of hexane. The precipitated homopolymer was vacuum filtered and dried in a vacuum oven at 40 °C. Finally, the solid was dissolved in water and placed in a dialysis membrane (MWCO: 14,000 Da). After a certain number of external water renewals, the aqueous solution was condensed in a rotary evaporator, followed by freeze drying, to finally obtain the homopolymer in its solid state.

### 2.3. Synthesis of P(DMAPMA-co-MMA_35_) Copolymers

In a three-necked 250 mL spherical flask, equipped with a magnetic stirrer, 12.7 mL of the monomer DMAPMA and 3.2 mL of the monomer MMA were placed in 75 mL of the solvent THF, and left under stirring and bubbling with nitrogen conditions for about 30 min. Next, 82.54 mg (0.5 mol % over the total monomer concentration) of AIBN initiator was added, and the system was stirred at 60 °C under a nitrogen atmosphere overnight. Next day, the copolymer was obtained through precipitation in hexane and filtration under vacuum. The product was dried in a vacuum pump at 40 °C. The same procedure with different monomer DMAPMA and MMA quantities was followed to synthesize the P(DMAPMA-co-MMA_20_) copolymer.

### 2.4. Synthesis of Alkylated Copolymer P(DMAPMA-co-MMA35) with 8.1% Dodecyl Bromide

In a 100 mL spherical flask, equipped with a magnetic stirrer, 2.5 g of P(DMAPMA-co-MMA35) copolymer was placed in 50 mL THF. The mixture was left under stirring conditions until the polymer was dissolved (~30 min). Nitrogen bubbling was applied, and then 0.33 mL of 1-bromododecane was added. The system was stirred at a temperature of 70 °C and under a nitrogen atmosphere for 72 h. In the next step, precipitation in petroleum ether was carried out, followed by vacuum filtration and washing with petroleum ether. The precipitate was then dried in a vacuum pump at 40 °C.

In a similar way, several alkylated DMAPMA-based were also produced using various bromoalkanes like 1-bromopropane, 1-dodecyl bromide, and 1-bromo-decahexane.

### 2.5. Characterization of Copolymers

The synthesized polymers were characterized, in terms of their composition, by proton nuclear magnetic resonance (^1^H-NMR) spectroscopy at 25 °C on a Bruker Advance DPX spectrometer at 400 MHz (Billerica, MA, USA). Deuterated solvents D_2_O and DMSO-d_6_ were used to dissolve the samples, while tetramethylsilane (TMS) was used as an internal standard. Attenuated total reflection–Fourier transform infrared (ATR-FTIR) spectra of the polymers were recorded on a Bruker Platinum ATR-FTIR spectrometer (Billerica, MA, USA).

ζ-potential and particle size studies of the materials were performed using the Malvern Zetasizer Nano-ZS device (Malvern Instruments Ltd., Worcestershire, UK). The light source was a 4 mW He-Ne laser at 633 nm, while the intensity of the scattered light was measured at 173°. In all measurements, the temperature was adjusted from 22 to 70 °C. The solutions, after being filtered, were placed in a DTS 1070 cell (Malvern Instruments Ltd., Worcestershire, UK). Transmission electron microscopy (TEM) was used to observe the formed polymer nano-assembles. For this purpose, a JEM-2100 microscope (JEOL, Tokyo, Japan) operating at 200 kV was used. Four microliters of aqueous polymer solution (at a polymer concentration of 1% *w*/*v*) was deposited on TEM carbon grids. The grids were left at room temperature until the full evaporation of water.

### 2.6. Thermo-Responsive Properties

The optical density of aqueous solutions of all polymers at temperatures from 25 to 80 °C was determined using a HITATCHI UV-Vis U-1800 spectrophotometer (Hitachi, Schaumburg, IL, USA), which was equipped with a suitable thermostatic quartz cell holder, with 1 cm optical path. The optical density values were taken at 500 nm. The temperature was regulated by a thermostatic water bath that delivered water through insulated tubes to the cell.

### 2.7. pH Adjustment

Aqueous polymer solutions with pH values set at 12, 13, and 14 were prepared by dissolving the polymers in sodium hydroxide aqueous solutions of 0.01, 0.1, and 1 M, respectively. The aqueous 1% *w*/*v* polymer solution has a pH~11. For the less basic solutions, monopotassium phosphate (KH_2_PO_4_) buffer solutions were prepared upon the addition of adequate volumes of NaOH 0.1 M. For more concentrated polymer solutions, it was observed that the buffer capacity was not sufficient, and pH increased relatively to the pH of the buffer solution.

### 2.8. CO_2_ Sensitivity

Three aqueous P(DMAPMA-co-MMA_35_) polymer solutions at a final volume 20 mL and concentration 0.1% *w*/*v* were prepared. The first solution was not treated with CO_2_. In the second solution, CO_2_ bubbling was carried out for 1 h. The third solution was bubbled the solution with CO_2_ for 1 h and then bubbling it with Ar, under heat (~50 °C), for 1 h.

### 2.9. Determination of Critical Aggregation Concentration (CAC)

For the physicochemical study of polymer solutions, the fluorescent Nile Red dye was used as a probe. Emission spectra of Nile Red were obtained using a PERKIN ELMER model LS 45 (Perkin Elmer, Luminescence Spectrometer, PERKIN ELMER, Waltham, MA, USA). Into 3 mL of the aqueous polymer samples, 5 µL of a Nile Red solution in THF (10^−3^ M) was added using a micropipette. The samples were then placed in a quartz cuvette (Quartz Fluorimeter Cuvette, Teflon Cover, 10 mm Light Path, Fisher Scientific, Thessaloniki, Greece). The emission scan was performed within a range of 560 nm to 700 nm with a scan speed of 200 nm/min, while the excitation wavelength was 550 nm.

### 2.10. Measurement of Total Organic Carbon (TOC) and Total Nitrogen (TN)

To determine CO_2_ adsorption by P(DMAPMA-co-MMA_35_) copolymer, total organic carbon (TOC) and total nitrogen (TN) were measured. TOC and TN analyses were performed using the Shimadzu TOC analyzer (TOC-VCSH), connected to a chemiluminescence detector (TNM-1).

### 2.11. Antimicrobial Properties

#### 2.11.1. Bacterial Culture Preparation

*Escherichia coli* (*E. coli*, MC1061 strain, lab collection) and *Pseudomonas aeruginosa* (*P. aeruginosa*, NCTC 10662 strain, Health Protection Agency, Porton Down, Salisbury, UK) were used as representatives of Gram-negative organisms while *Staphylococcus aureus* (*S. aureus*, NCTC 6571 strain, Health Protection Agency, Porton Down, Salisbury, UK) was used as a representative Gram-positive organism to test the antimicrobial activity of the P(DMAPMA-co-MMA_35_) copolymer. Cultures to provide bacteria for viability assays were set in 8 mL of LB broth from single colonies and left for approximately 18 h (overnight) at 37 °C in 15 mL tubes placed horizontally at 80 rpm (final cell density of approximately 10^8^–10^9^ cfu·mL^−1^).

#### 2.11.2. Bacteria Cell Reduction

Initially, glass coupons (18 × 18 mm) were coated with polymeric solutions under sterile conditions and left to dry at room temperature (RT) overnight. Next, 20 μL aliquots of overnight cultures of each bacterial species were placed on each of the glass coupons coated with the polymer for 2 h at 22 °C. Afterwards each sample was transferred into a sterile 50 mL tube containing 30 mL of LB broth. The tubes were placed horizontally and were incubated at 80 rpm at 37 °C for 8 h for the *E. coli* and *P. aeruginosa* and 24 h for the *S. aureus* cultures. Cell growth (scattering) was measured at 600 nm, diluting in water if needed so that the final OD_600_ (optical density at 600 nm) remained ≤0.5. The volume of inoculation (20 μL) and the time of growth for each species were optimized to ensure that all growth measurements were performed when the *E. coli*, *P. aeruginosa*, and *S. aureus* controls (no polymer exposure) were in their exponential phase of growth. Each experiment was replicated on different days with bacteria from different starting cultures. The effect of polymers on cell reduction was estimated by comparing the growth of the control cells (without polymer coating) with that of cells exposed to the polymer, using the following equation:

$$Cell\;Reduction\;\%=\frac{{OD}_{600}Control-{OD}_{600}Sample}{{OD}_{600}Control}\times 100\%$$

## 3. Results and Discussion

### 3.1. Synthesis and Characterization of Polymers

**Scheme 1:**
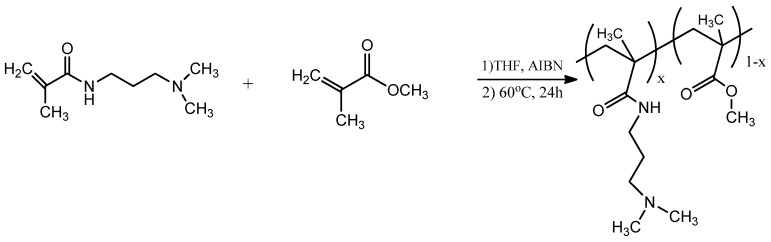
Synthesis of P(DMAPMA-co-MMAy) copolymers via free radical copolymerization in organic solvent.

Apart from the homopolymer PDMAPMA, two copolymers of DMAPMA with MMA were also prepared. In all cases, the synthesis was carried out in organic solvent using AIBN as initiator, as schematically presented in Scheme 1. The copolymers are denoted as P(DMAPMA-co-MMA_y_), where the suffix y refers to the molar percentage of MMA structural units, as determined through ^1^H-NMR characterization.

**Figure 1:**
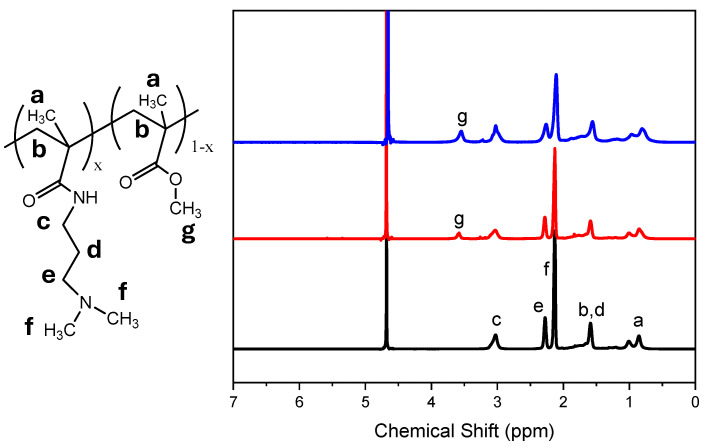
^1^H-NMR spectra of PDMAPMA homopolymer, P(DMAPMA-co-MMA_20_), and P(DMAPMA-co-MMA_35_) copolymers in D_2_O.

All synthesized polymers were soluble in water and characterized by ^1^H-NMR in D_2_O (Figure 1). No sharp peaks due to monomers were detected at 5.5–6.5 ppm, thus confirming the success of polymerization. The presence of the peaks at 0.8–1.8 ppm is due to the -CH_2_- (b) and -CH_3_ (a) groups of the main polymeric chain of the two structural units of the copolymer. 

The presence of DMAPMA was confirmed by the sharp peaks at 2.2 and 2.3 ppm corresponding to the two methyl groups -CH_3_ (f) and one group -CH_2_- (e), respectively, attached to the tertiary nitrogen, as well as the peak at 3.2 ppm corresponding to protons of the -CH_2_- group (c) attached to the amide group (-CONH-CH_2_-). Finally, the presence of MMA was confirmed by the peak at 3.6 ppm, which corresponded to the protons of the methyl group (-CH_3_, g) attached to the oxygen of the ester.

To determine the composition of the copolymers, the peaks at 3.6 ppm (g) and at 2.2 ppm (f) were used (Table 1). The results show that the MMA content of the copolymers is slightly higher, as compared to the feed composition.

The PDMAPMA homopolymer and P(DMAPMA-co-MMAy) copolymers were also characterized through ATR-FTIR and the results are shown in Figure S1.

### 3.2. pH- and Ionic Strength-Controlled Thermo-Responsiveness

**Figure 2:**
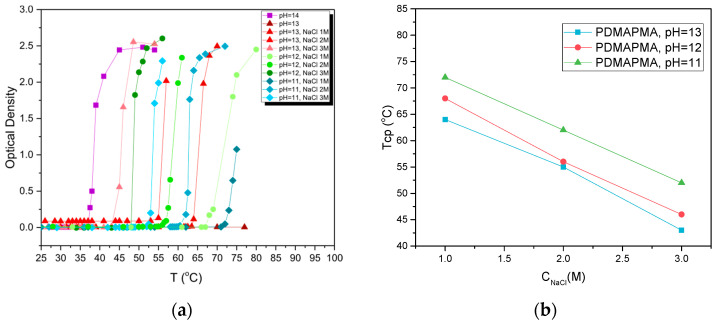
(**a**) Optical density measurements and (**b**) cloud point temperature of 1% *w*/*v* aqueous solutions of PDMAPMA homopolymer as a function of pH and concentration of NaCl.

As mentioned [58], the homopolymer PDMAPMA exhibits an LCST type phase separation at 35 °C only when the pH of the aqueous solution is very high (pH 14), in the absence of salt. This behavior is also verified in Figure 2a, where it can be seen that the optical density of the transparent at room temperature aqueous PDMAPMA solution at pH 14 increases abruptly upon heating above 37 °C. In contrast, the solution remains fully transparent within the whole temperature range studied (up to ~80 °C) when pH is less basic (pH 13).

The influence of ionic strength on the cloud point of the homopolymer at pH 11–14 was also investigated. The higher pH values (pH 12, 13, and 14) were achieved by using as solvent a NaOH solution of the respective nominal concentration (0.01 M, 0.1 M, and 1 M), while the value of pH 11 was obtained using pure water, since the polymer is weakly basic. To control the ionic strength, NaCl was added into the solvent at the desired concentration. The dependence of the cloud points on salt concentration, as determined from the data shown in Figure 2a, is presented in Figure 2b. It is worth noting that, in the presence of salt, a salting out effect is observed, where the homopolymer presents LCST behavior at much less basic solutions, indicating the kosmotropic effect of salt in our case, as found also in similar systems [60]. At the same salt concentration, the cloud point increases with decreasing pH. Moreover, with increasing salt concentrations, Tcp occurs at lower temperatures following a practically linear dependence. This allows us to cover the entire temperature range of usual practical interest (from room temperature up to 80 °C) just by selecting the desired salt concentration.

**Figure 3:**
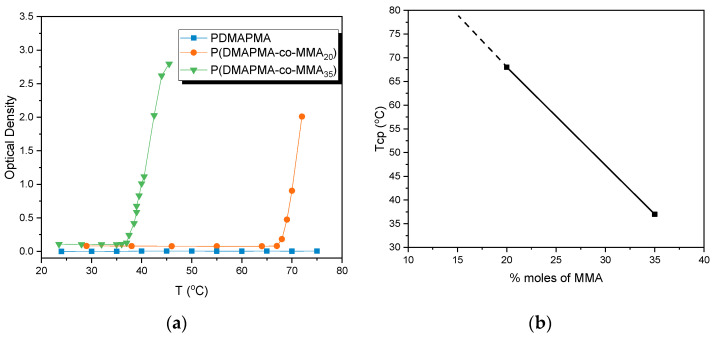
(**a**) Optical density measurements of aqueous solutions of DMAPMA-based polymers and (**b**) cloud point temperature as a function of MMA content (% mol).

The incorporation of hydrophobic MMA units in the polymer chain greatly influences the LCST behavior of these systems, as seen in Figure 3. These studies were performed in pure aqueous solutions (pH 11). From optical density changes with increasing temperature (Figure 3a), it can be seen that, unlike the homopolymer, the copolymers turn very cloudy at a temperature dependent on the MMA content. The cloud point temperature of the aqueous polymer solutions as a function of the % molar composition in the hydrophobic MMA monomer is displayed in Figure 3b. As can be seen, the incorporation of 20% hydrophobic MMA units into the P(DMAPMA-co-MMA20) chains affects the hydrophilic/hydrophobic balance, making the copolymer more hydrophobic and leading to the appearance of Tcp at ~67 °C. Moreover, as the hydrophobic MMA content increases from 20 to 35% mol, the Tcp decreases by about 30 °C, leading to a Tcp of ~37 °C.

**Figure 4:**
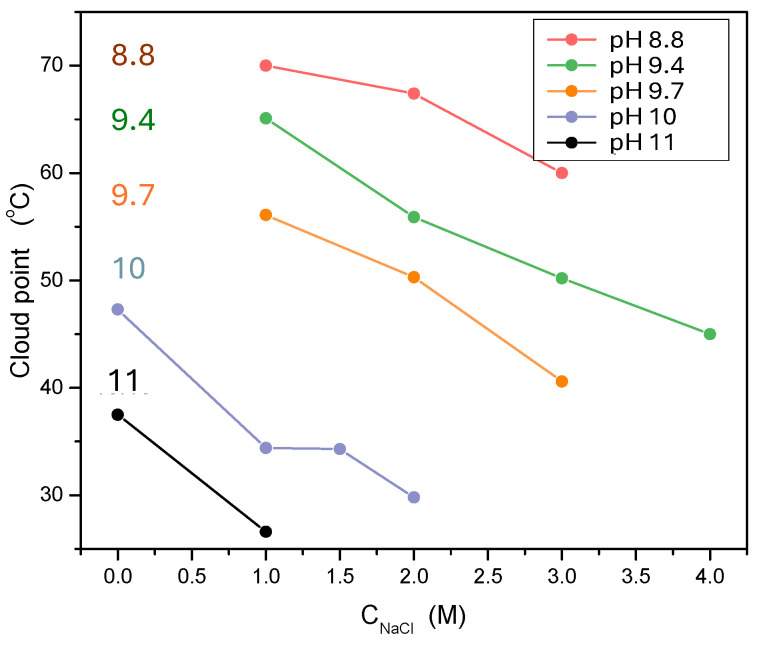
Plot of cloud point temperature (Tcp) of aqueous solutions of copolymer P(DMAPMA-co-MMA_35_) at different pH as a function of salt concentration.

To further explore the effect of pH and salt on the thermo-responsiveness, the copolymer P(DMAPMA-co-MMA_35_) was chosen. For this study, diluted KH_2_PO_4_ buffers were used to adjust pH, while NaCl was used to control the ionic strength. Due to the weak basic character and the considerable concentration (1% *w*/*v*) of the copolymer, the buffering capacity of the buffers used was not sufficient and the pH of the final solutions was verified using a pH meter (Table S1). The variations in the cloud point with the NaCl concentration at these pH values for the aqueous P(DMAPMA-co-MMA_35_) solutions are presented in Figure 4. It is interesting to note that this copolymer presents an LCST behavior at ~47 °C in pure buffer solution even at pH 9.9, much lower than the pH 14 value required for the homopolymer. Moreover, the LCST behavior can be observed even at pH 8.7 in the presence of salt, namely when a large proportion of the DMAPMA units are charged, since the pKa value of DMAPMA is estimated to be 9.2 [63]. Finally, in agreement with the previous results, the cloud point appears at higher temperatures upon decreasing pH, while for each system, the cloud point decreases with increasing the NaCl concentration.

### 3.3. Self-Assembly of Polymers in Aqueous Solution

Previous studies suggest that the homopolymer PDMAPMA, unless under strongly basic conditions, is quite hydrophilic. Consequently, the introduction of the hydrophobic MMA units in the P(DMAPMA-co-MMA_y_) copolymers is expected to lead to amphiphilic materials. Thus, at a next step, the potential self-organization of the (co)polymers was examined through Nile Red probing. Nile Red, a widely used dye for staining purposes in biology and in the characterization of disperse systems such microemulsions, polymer colloids, host–guest and polymer/surfactant systems, is a slightly water-soluble probe [60,64]. Thus, it exhibits a strong fluorescence when dissolved in hydrophobic (micro)environments, while it is non-fluorescent in water. As an example, the emission spectra of Nile Red in P(DMAPMA-co-MMA_35_) copolymer solutions for different copolymer concentrations (0.001% *w*/*v* to 1% *w*/*v*) at room temperature are presented in Figure S2. Upon excitation at 550 nm, an emission band centered at 625 nm is observed at higher copolymer concentrations. This emission is enhanced by increasing the concentration of the copolymer. This enhancement apparently indicates the solubilization of the dye in a more hydrophobic nonpolar microenvironment, originating from the organization of the copolymer in amphiphilic microstructures at higher concentrations.

**Figure 5:**
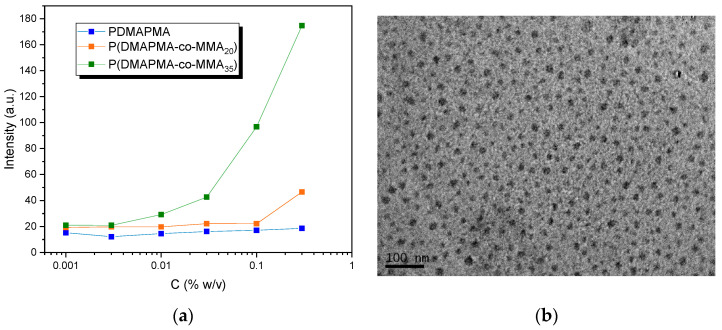
(**a**) Maximum intensity of emission spectra of Nile Red (centered at ~630 nm) in aqueous solutions of PDMAPMA homopolymer (blue), P(DMAPMA-co-MMA_20_) copolymer (orange), and P(DMAPMA-co-MMA_35_) copolymer (green), as a function of polymer concentration. (**b**) TEM image of P(DMAPMA-co-MMA_35_) copolymer at 1% *w*/*v*, in water (above CAC).

The maximum emission intensity of Nile Red is plotted in Figure 5a as a function of polymer concentration. Here, the results of the PDMAPMA homopolymer as well as the P(DMAPMA-co-MMA_y_) copolymers are given. As expected, Nile Red did not exhibit any fluorescence variation for all PDMAPMA concentrations studied, indicating that the homopolymer does not have the tendency to self-organize in any nanostructure. However, it is clear that this is not the case for the two copolymers. For both P(DMAPMA-co-MMA_y_) copolymers, the maximum emission intensity of Nile Red is low at lower polymer concentrations while it increases abruptly above a certain polymer concentration. This concentration can be considered the critical aggregation concentration (CAC), namely the concentration above which the polymer chains form self-associated structures. It can be observed that the MMA content affects the value of CAC, since it shifts the hydrophilic/hydrophobic balance towards hydrophobicity. Thus, the CAC value for the P(DMAPMA-co-MMA_20_) copolymer with 20% mol MMA content is detected at ~0.1% *w*/*v*; conversely, when the hydrophobic MMA content increases to 35% mol for the P(DMAPMA-co-MMA_35_) copolymer, the CAC value is found at a much lower polymer concentration, ~0.003% *w*/*v*.

TEM verified the Nile Red probing findings. The TEM image of P(DMAPMA-co-MMA35) copolymer, obtained at a polymer concentration much above CAC, is shown in Figure 5b, where micelle-like structures with an average size of about 15 nm are observed.

**Figure 6:**
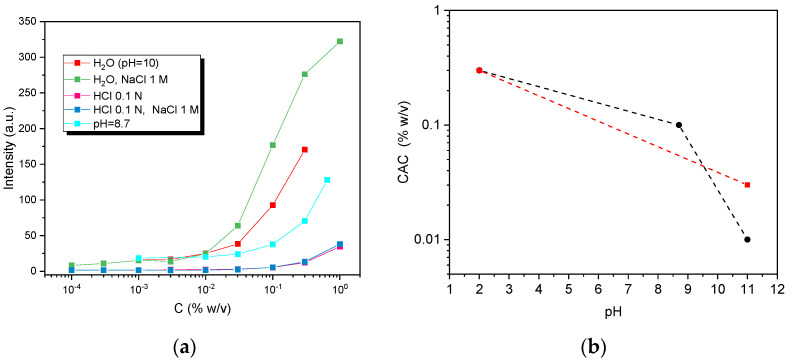
(**a**) Maximum intensity of emission spectra of Nile Red (centered at ~630 nm) in aqueous solutions of the P(DMAPMA-co-MMA_35_) copolymer at different pH values and in the presence of absence of NaCl, as a function of polymer concentration. (**b**) CAC versus pH in the absence (black line) or presence (red line) of NaCl.

The self-assembly study of the DMAPMA-based polymers was further studied not only in pure aqueous solution but also at different pH values and in the presence of NaCl 1 M. In Figure 6a,b, it is observed that when the copolymer P(DMAPMA-co-MMA_35_) is dissolved in water (pH 10), CAC appears at a concentration of 0.03% *w*/*v*, while in the presence of salt, CAC appears at a somewhat lower concentration, ~0.01% *w*/*v*, while the fluorescence signal is larger. When the polymer is dissolved in 0.1 M HCl, CAC is observed at a much higher concentration, close to 1% *w*/*v*, without any difference in behavior, even in the presence of 1 M NaCl. Finally, at the intermediate pH value, pH 8.7, the CAC appears at a concentration of 0.1% *w*/*v*.

### 3.4. Study of CO_2_-Sensitivity

**Figure 7:**
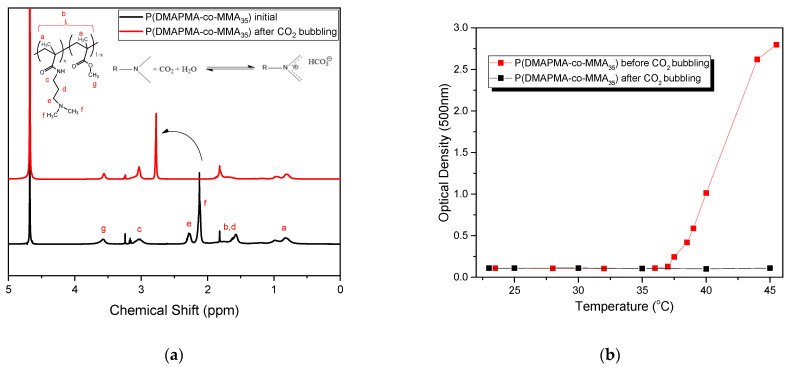
(**a**) ^1^H-NMR spectra of P(DMAPMA-co-MMA_35_) copolymer in D_2_O solvent before and after CO_2_ treatment; (**b**) optical density measurements of the copolymer P(DMAPMA-co-MMA_35_) before and after the treatment of the polymer solution with CO_2_.

Because of the weak basic character of the (co)polymers, their aqueous solutions are expected to adsorb CO_2_ through an acid–base reaction (see inset of Figure 7). To explore this phenomenon, a solution of P(DMAPMA-co-MMA_35_) copolymer in D_2_O was bubbled with CO_2_ and characterized through ^1^H-NMR spectroscopy. The ^1^H-NMR spectra of this solution, before and after CO_2_ bubbling, are shown in Figure 7a. As can be seen, after CO_2_ treatment, the peaks of –CH_2_–N–(CH_3_)_2_ and –CH_2_–CH_2_–N(CH_3_)_2_ shift from 2.3, 2.2, and 1.6 ppm to 3.0, 2.8, and 1.8 ppm, respectively, reflecting the protonation of the tertiary amine group.

**Figure 8:**
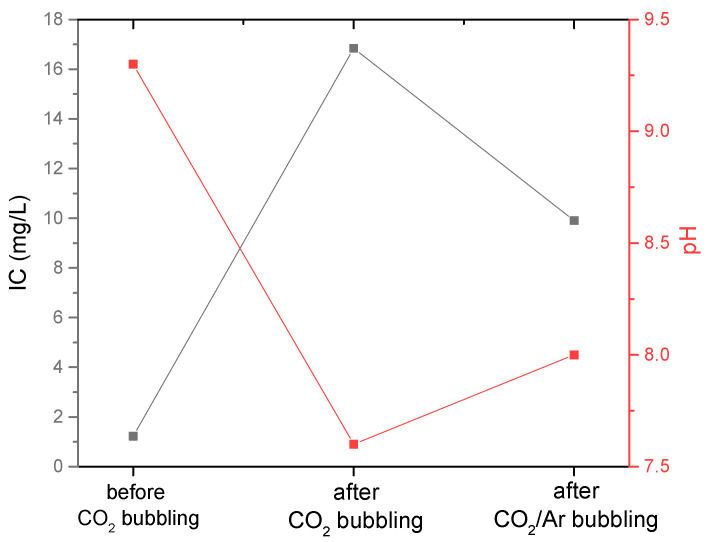
Variation in the inorganic carbon (IC) (mg/L) content and pH of the aqueous P(DMAPMA-co-MMA_35_) solutions, before and after CO_2_ bubbling, followed by Ar bubbling. The polymer concentration is 0.1% *w*/*v*.

At the next step, a turbidimetry study was performed using an aqueous solution of the copolymer after CO_2_ treatment to check any variation in the thermal response (Figure 7b). It should be noted that, before CO_2_ bubbling, the aqueous polymer solution exhibited a cloud point temperature of 37 °C. In contrast, after the supply of CO_2_, the solution did not exhibit any turbidity up to 45 °C, as seen in Figure 8. Apparently, the protonation of the tertiary amine groups turns the copolymer into a polyelectrolyte, thus losing its thermo-responsive character.

To better quantify this behavior, three aqueous solutions of P(DMAPMA-co-MMA_35_) polymer were prepared, with a concentration of 0.1% *w*/*v*. The first solution was not treated with CO_2_. In the second solution, CO_2_ bubbling was carried out for 1 h. The third solution was bubbled with CO_2_ for 1 h and then bubbled with Ar under heating conditions for 1 h. The three solutions were characterized through conductivity, pH, total organic carbon/inorganic carbon, and total nitrogen (TOC/TN) determination. The results are presented in Table 2 and in Figure 8.

As expected, the untreated solution (first solution) presents a low conductivity and a high pH value, since the polymer is mostly under the uncharged weakly basic form. The inorganic content, IC, of this solution is marginal and it can be attributed to the slight adsorption of atmospheric CO_2_. Considering that each structural DMAPMA unit contains two nitrogen atoms, the IC/TN ratio suggests that just a small portion of the tertiary amine groups, less than 4% mol (taking into account that only half of nitrogen atoms of the copolymer correspond to the amine groups), is neutralized by carbonate ions, originating from atmospheric CO_2_. In contrast after CO_2_ bubbling (second solution), the conductivity of the solution increases significantly, while pH decreases, as a consequence of the CO_2_ adsorption and the neutralization of tertiary amine groups. The increased IC value suggests that the copolymer has now captured significant CO_2_ amounts. In fact, the molar IC/TN ratio indicates that about half of the tertiary amine groups are now neutralized. After Ar bubbling (third solution), the conductivity decreases again, while pH increases. However, conductivity and pH do not reach the initial values of the first solution, indicating that this treatment is not sufficient to restore the polymer to its original fully uncharged state. In fact, IC is still important, while the IC/TN ratio suggests that a quite large proportion of tertiary amine groups (~30% mol) remain neutralized by carbonate ions (originating from CO_2_ bubbling). This is a limitation of our present materials, possibly due to the rather high basicity of DMAPMA units, leading to pH~9.3. In combination with the fact that the pK_2_ of carbonate is ~10 [65], an important fraction of CO_2_ is adsorbed as CO_3_^2−^ anions. The combination of a higher temperature upon Argon flushing with a slightly lower initial pH could possibly lead to better reversibility.

### 3.5. Alkylation and Antimicrobial Properties

A common class of polymeric biocide is based on quaternary ammonium compounds (QACs). The effectiveness of these polymers is affected by the charge density of the polymer and the alkyl chain length of the cationic moieties. Longer alkyl chains enhance antibacterial activity but may lead to issues such as polymer aggregation and increased hemolytic activity [66]. The balance between cationic and hydrophobic components in the polymer structure is critical for optimal biocidal activity. On the other hand, excessive hydrophobicity results in high toxicity and poor solubility, while too little hydrophobicity weakens antibacterial efficacy and may cause erythrocyte aggregation. The basic principle is to include the minimum amount of hydrophobic content necessary for effective antibacterial activity.

**Scheme 2:**
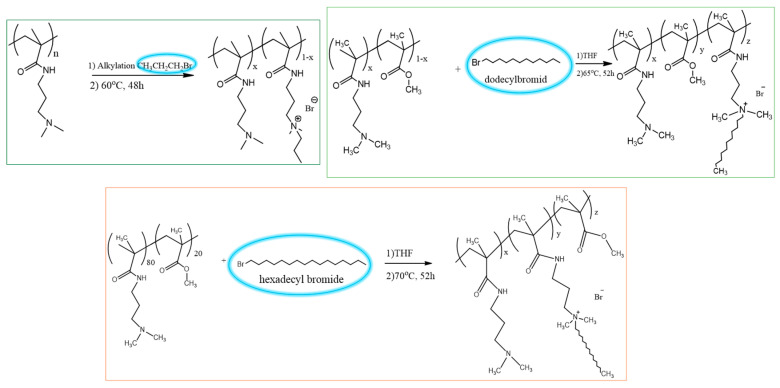
Alkylation of PDMAPMA homopolymer with 1-bromopropane, and P(DMAPMA-co-MMAy) copolymers with 1-dodecyl bromide and 1-bromo-decahexane.

Thus, in this study, several alkylation attempts were carried out (Scheme 2). The synthesized polymers, both the PDMAPMA homopolymer and P(DMAPMA-co-MMAy) copolymers, have tertiary amine groups and amide groups. It was expected that the brominated alkanes would react with the tertiary amine groups to form quaternary ammonium salts, since the amide groups were less reactive [67]. First, the alkylation of PDMAPMA homopolymer with 1-bromopropane was performed. Thus, it was possible to add a short chain length to the polymer chain at a relatively large proportion. Furthermore, the copolymers were alkylated with longer chains of 1-dodecyl bromide and 1-bromo-decahexane, aiming at the development of effective antimicrobial materials. However, due to the hydrophobicity of the molecule, the polymers were alkylated to a lesser extent with the longer alkyl chains in order to maintain the water-solubility of the products.

The materials were characterized by ^1^H-NMR spectroscopy, through which it was possible to identify the alkyl peaks and determine the alkylation degree. The ^1^H-NMR spectra along with the peaks’ assignment are presented in Figures S3–S5. The characterization results of the alkylated polymers after ^1^H-NMR spectroscopy are summarized in Table 3.

The alkylated copolymers were also characterized through ATR-FTIR and the results are shown in Figure S5.

**Figure 9:**
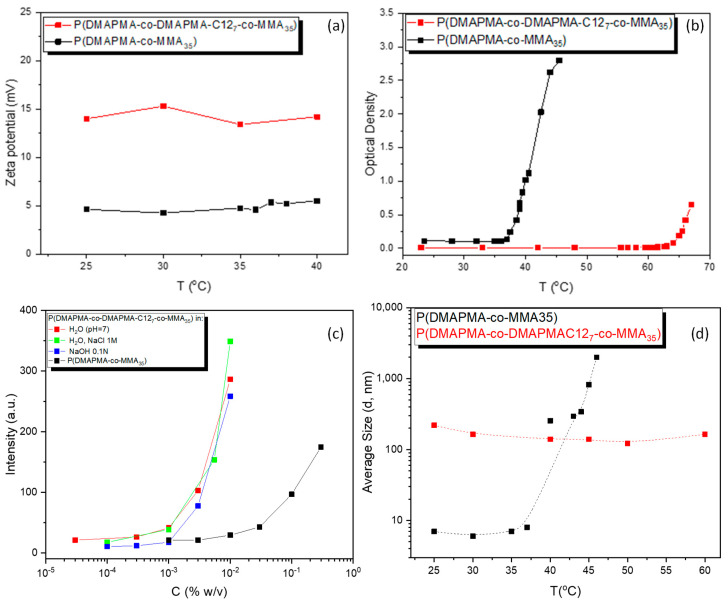
Determination of (**a**) ζ-potential and (**b**) optical density measurements of P(DMAPMA-co-MMA_35_) and alkylated P(DMAPMA-co-DMAPMAC12_7_-co-MMA_35_) copolymer aqueous solutions as a function of temperature. (**c**) Maximum intensity of emission spectra of Nile Red at 600–650 nm in aqueous solutions of P(DMAPMA-co-MMA_35_) and alkylated P(DMAPMA-co-DMAPMAC12_7_-co-MMA_35_) copolymers, as a function of polymer concentration. (**d**) Particle size distribution of P(DMAPMA-co-MMA_35_) and alkylated P(DMAPMA-co-DMAPMAC12_7_-co-MMA_35_) copolymers as a function of temperature. The dashed lines are guides for the eye.

The behavior of the alkylated products in aqueous solution was investigated through a variety of techniques, namely turbidimetry, ζ-potential, dynamic light scattering, and Nile Red probing. The behavior of the alkylated copolymer P(DMAPMA-co-DMAPMAC12_7_-co-MMA_35_) is compared in Figure 9, with that of the mother copolymer P(DMAPMA-co-MMA_35_). As expected, the non-alkylated P(DMAPMA-co-MMA_35_) copolymer exhibits a slightly positive ζ-potential (Figure 9a), due to the slight protonation of the weakly basic tertiary amine groups, throughout the whole temperature range studied (25–40 °C). In contrast, the ζ-potential of the alkylated P(DMAPMA-co-DMAPMAC12_7_-co-MMA_35_) product is remarkably high, since a significant number of the amine groups are now quaternized.

The variations in the temperature of the aqueous solution turbidity of the alkylated P(DMAPMA-co-DMAPMA-C12_7_-co-MMA_35_) copolymer at pH 14 is compared with the respective behaviors of aqueous P(DMAPMA-co-MMA_35_) solution (pH 11) in Figure 9b. As a consequence of the high positive charge, even under these strongly alkaline conditions (where the P(DMAPMA-co-MMA_35_) copolymer is not soluble), the alkylated copolymer is soluble at low temperatures and forms a cloud point at a rather high temperature, around 64 °C. In fact, it was verified that at a lower pH the alkylated product remained fully soluble across the whole temperature range studied.

As already shown in Figure 5a, the CAC of P(DMAPMA-co-MMA_35_) copolymer (aqueous solution) was found to be ~0.03% *w*/*v*. Nile Red probing was used to study also the self-association behavior of the alkylated P(DMAPMA-co-DMAPMA-C12_7_-co-MMA_35_) copolymer (Figure 9c). The study was performed in water (pH 7), in acidic (HCl 0.1 M) and alkaline conditions (NaOH 0.1 M), and in the presence of salt (NaCl 1 M). In all conditions, the alkylated copolymer exhibited a CAC at the same concentration of 0.001% *w*/*v*. Obviously, the alkylation of P(DMAPMA-co-MMA_35_) enhanced the hydrophobic character of the copolymer, which then displayed a decreased CAC compared to the higher CAC of the P(DMAPMA-co-MMA_35_) precursor (~0.03% *w*/*v* at pH 11).

The temperature-induced changes in the size of the thermo-responsive P(DMAPMA-co-MMA_35_) and P(DMAPMA-co-DMAPMAC12_7_-co-MMA_35_) polymers were followed by DLS. The variations in the temperature of the size distributions of the copolymers are shown in Figures S7 and S8 and the results are given in Figure 9d. Below 35 °C, the P(DMAPMA-co-MMA_35_) chains probably assemble in unimers with sizes of ~6–8 nm in diameter. Above 35 °C, the solution becomes cloudy and copolymer aggregates with higher sizes are observed. These aggregates exhibit a tendency to increase in size with increasing temperature. Similar results were reported in a previous study concerning poly(di(ethylene glycol) methyl ether methacrylate-co- N,N-dimethylacrylamide) (P(DEGMA-co-DMAM)) statistical polymers [52]. In agreement with the turbidity study, the self-assembly of the alkylated P(DMAPMA-co-DMAPMAC12_7_-co-MMA_35_) product, on the other hand, seems to be unaffected by the temperature variation since the average size of the polymer nano-assemblies is constant (somewhat less than 200 nm), with no change observed throughout the temperature range studied. In this case, alkylated groups are organized into hydrophobic domains mostly due to the amphiphilic nature of the copolymer rather than hydrogen bonding with water molecules [53].

**Figure 10:**
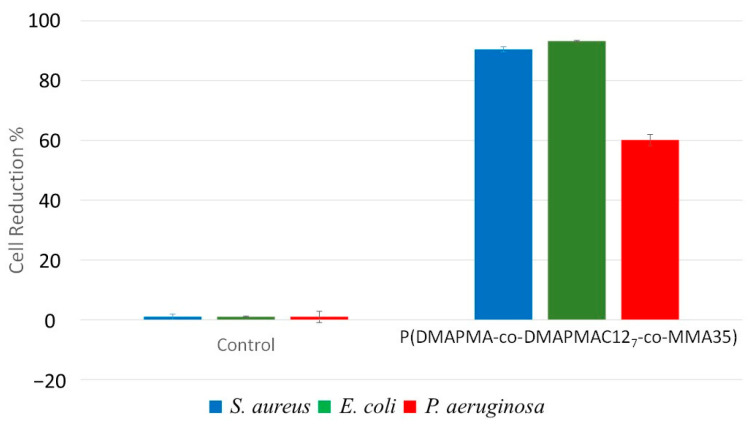
% Cell reduction in three bacteria: *S. aureus*, *E. coli*, and *P. aeruginosa* after contact for 120 min with the alkylated copolymers P(DMAPMA-co-DMAPMAC12_7_-co-MMA_35_).

In order to explore the potential antimicrobial ability of DMAPMA-based polymers, we performed an initial study of the effect of the alkylated P(DMAPMA-co-DMAPMAC12_7_-co-MMA_35_) copolymer towards various bacteria. In Figure 10, the % cell reduction in three common bacteria (i.e., *S. aureus*, *E. coli*, and *P. aeruginosa*) is presented after contact of bacteria for 120 min with P(DMAPMA-co-DMAPMAC12_7_-co-MMA_35_). As can be seen, P(DMAPMA-co-DMAPMAC12_7_-co-MMA_35_) exhibited different activities against *S. aureus*, *E. coli*, and *P. Aeruginosa*, after two hours of contact. More specifically, the polymer was quite active against *S. aureus* (85.2% cell reduction) and even more active against *E. coli* (94.3% cell reduction). The antimicrobial efficacy, however, against *P. aeruginosa*, was moderated and a cell reduction of 73.8% was found. This is attributed to the fact that *P. aeruginosa* has significantly lower outer membrane permeability (between 12 and 100 times less than that of *E. coli*) which contributes to its higher resistance [68]. Achieving the optimal balance between the positively charged DMAPMAC12 units and the non-alkylated DMAPMA units in the copolymers is the key to providing high antibacterial efficacy, while preserving multifunctionality and responsiveness.

## 4. Conclusions

The multifunctional characteristics of copolymers based on the pH-sensitive DMAPMA unit and the hydrophobic MMA unit were explored in the present work. It was found that while the homopolymer PDMAPMA presents an LCST-type behavior only at extreme pH values (pH 14), the introduction of the hydrophobic MMA units shifts this thermo-sensitivity towards more accessible pH values (down to pH~11 in pure water and pH ~8.5 in the presence of salt). This change becomes more important as the hydrophobic MMA content of the copolymers increases. In addition, the introduction of MMA units leads to amphiphilic systems that self-assemble in water, organized in nanostructures with a size of ~15 nm (for P(DMAPMA-co-MMA_35_)) above CAC. Finally, such copolymers exhibit visual CO_2_-sensig abilities, since the LCST behavior is sensitive to the reaction of the weakly basic amine groups of DMAPMA units with CO_2_ in water. This property was explored through a combination of ^1^H NMR, turbidimetry, and TOC/TN. The partial reversibility of the reaction is a drawback of the copolymers discussed in the present work and should be effectively addressed through systematic optimization. The combination of a higher temperature after argon flushing with a slightly lower initial pH could possibly lead to a better reversibility. However, to maintain thermoresponsiveness in this slightly more acidic solution, alternative copolymers, for example, with higher MMA contents or more hydrophobic monomers, should possibly be designed.

The partial alkylation of the copolymers leads to amphiphilic products. For example, the copolymer P(DMAPMA-co-DMAPMAC12_7_-co-MMA_35_) is effectively stabilized in water as a larger aggregate (~200 nm) over a wide temperature range due to the increased ζ-potential (+15 mV), as compared to the unmodified precursor. Such products have proven to be quite efficient biocides for microorganisms such as *E. coli* and *S. aureus*, indicating the potential of these materials for antimicrobial applications.

In conclusion, a novel family of DMAPMA-based copolymers was developed in the present work, exhibiting thermosensitivity combined with functionalities related to the properties of the tertiary amine group, such as pH-responsiveness, CO_2_-senstivity, and the biocidal activity of alkylated amines. Although the design was based on MMA as a comonomer in this work, alternative, more or less hydrophobic, comonomers like methacrylamide ones could also be exploited, enabling the more efficient control of the properties and the optimization of the systems, depending on the desired application. Along with the other properties of the DMAPMA monomer, these materials could offer attractive alternatives for the design of highly valuable systems, such as smart devices in technological and biomedical fields. 

## Figures and Tables

**Table 1:** ^1^H-NMR characterization of the synthesized (co)polymers.

Polymer	Feed Composition (% mol DMAPMA)	Feed Composition (% mol ΜΜA)	^1^H-NMR Results (% mol MMA)
PDMAPMA	100	0	0
P(DMAPMA-co-MMA_20_)	85	15	20
P(DMAPMA-co-MMA_35_)	70	30	35

**Table 2:** Results of measurements of conductivity (μS/cm^−1^), pH, total organic carbon (TOC, mg/L), total carbon (TC, mg/L), inorganic carbon (IC, mg/L), and total nitrogen (TN, mg/L).

A/A	Conductivity (μS/cm^−1^)	pH	TOC (mg/L)	TC (mg/L)	IC (mg/L)	TN (mg/L)	Molar IC/TN Ratio
1st	29	9.31	400.9	402.2	1.222	77.32	0.018
2nd (CO_2_)	166.4	7.62	411.6	428.4	16.84	81.56	0.24
3rd (CO_2_ + Ar)	96.6	8.02	395.7	405.6	9.913	75.60	0.15

**Table 3:** Characterization of all alkylated polymers.

Polymer	Feed (% Moles in MMA)	^1^H NMR (% Moles in MMA)	Solubility in Water
P(DMAPMA-co-DMAPMA-C3_48_)	52.0	48.0	✓
P(DMAPMA-co-DMAPMA-C12_16_-co-MMA_20_)	16.3	16.0	✓
P(DMAPMA-co-DMAPMA-C16_9_-co-MMA_20_)	8.7	9.0	✓
P(DMAPMA-co-DMAPMA-C12_15_-co-MMA_35_)	16.0	15.0	x
P(DMAPMA-co-DMAPMA-C12_7_-co-MMA_35_)	8.1	7.0	✓

## Data Availability

Data are contained within the article or Supplementary Materials.

## Supplementary Materials

The following supporting information can be downloaded at: https://www.mdpi.com/article/10.3390/polym17141896/s1. Figure S1. (a) ATR-FTIR spectra of PDMAPMA and PMMA homopolymers, the P(DMAPMA-co-MMAy) copolymers and a mixture of the liquid monomers containing 35%mol MMA: (b) a magnification of the wavenumber area 1000–2000 cm^−^1 of Figure S1a; Table S1. pH values of the prepared buffer solutions and pH values of each solution after the addition of the copolymer P(DMAPMA-co-MMA_35_) at a concentration of 1% *w*/*v*; Figure S2. Emission spectra of Nile Red at different P(DMAPMA-co-MMA_35_) polymer concentrations; Figure S3. ^1^H-NMR spectra in D_2_O of the PDMAPMA homopolymer in combination with the 1-bromopropane alkylated polymer P(DMAPMA-co-DMAPMA-C3_48_); Figure S4. ^1^H-NMR spectra in D_2_O of the P(DMAPMA-co-MMA_20_) copolymer (blue curve) in combination with the 1-bromododecane alkylated polymers (green curve) P(DMAPMA-co-DMAPMA-C12_16_-co-MMA_20_) and 1-bromodecahexane (red curve) P(DMAPMA-co-DMAPMA-C16_9_-co-MMA_20_); Figure S5. ^1^H-NMR spectra in DMSO of the copolymer P(DMAPMA-co-MMA_35_) (blue curve) in combination with the alkylated polymers with 7% 1-bromododecane (green curve) P(DMAPMA-co-DMAPMA-C12_7_-co-MMA_35_) and 15% (orange curve) P(DMAPMA-co-DMAPMA-C12_15_-co-MMA_35_); Figure S6. ATR-FTIR spectra of the alkylated polymers; Figure S7. Intensity weighted particle size distribution of 1% *w*/*v* non-alkylated P(DMAPMA-co-MMA35) copolymer aqueous solutions obtained from DLS measurements, at different temperatures; Figure S8. Intensity weighted particle size distribution of 1% *w*/*v* alkylated P(DMAPMA-co-DMAPMAC12_7_-co-MMA_35_) copolymer aqueous solutions obtained from DLS measurements, at different temperatures. Reference [69] is cited in the supplementary materials.
