# Supplementary material for: Multifunctional Amphiphilic Biocidal Copolymers Based on N-(3-(Dimethylamino)propyl)methacrylamide Exhibiting pH-, Thermo-, and CO2-Sensitivity
Source: Polymers (Basel). 2025 Jul 9;17(14):1896. doi: 10.3390/polym17141896 (PMC12299032; doi:10.3390/polym17141896)
Supplement: Supplementary file 1 [file polymers-17-01896-s001.zip › polymers-3690820-supplementary.pdf]

Supplementary Material

Multifunctional Amphiphilic Biocidal Copolymers Based on N-(3-(Dimethylamino)propyl)methacrylamide Exhibiting pH-, Thermo-, and CO_2-Sensitivity

Maria Filomeni Koutsougera [1], Spyridoula Adamopoulou [1], Denisa Druvari [1], Alexios Vlamis-Gardikas [1], Zacharoula Iatridi [2,*] and Georgios Bokias [1,3]

[1] Department of Chemistry, University of Patras, GR-26504 Patras, Greece; marifili.koutsougera@gmail.com (M.F.K.); up1064176@upnet.gr (S.A.); druvari@upatras.gr (D.D.); avlamis@upatras.gr (A.V.-G.); bokias@upatras.gr (G.B.)

[2] Department of Materials Science, University of Patras, GR-26504 Patras, Greece

[3] Foundation for Research and Technology-Hellas, Institute of Chemical Engineering Sciences (FORTH/ICE-HT), Stadiou Street, GR-26504 Patras, Greece

* Correspondence: iatridi@upatras.gr

Figure S1 shows the ATR-FTIR spectra of the PDMAPMA homopolymer and the P(DMAPMA-co-MMA_y) copolymers. For reasons of comparison, the spectra of PMMA homopolymer as well as of a mixture of the liquid monomers containing 35%mol MMA, are included for reasons of comparison.

In the spectra of the copolymers (**Figure S1 a**), the structural units of both homopolymers, PDMAPMA and PMMA, are clearly identified (for example, the peak at 1720 cm^{-1}, corresponding to the stretching vibration of the carbonyl group (C-O) of MMA and peaks at 3350, 1635 and 1520 cm^{-1} corresponding to N-H stretching and the amide I and amide II peaks of DMAPMA, respectively [1]. The asymmetric stretching peak appeared at 1150 cm^{-1}, which is attributed to the C-O-C group, is present both in the homopolymer as well as in the copolymers. It can be observed that the intensity of this peak is enhanced by increasing the MMA content in the copolymer. Likewise, if we compare the peak at 1720 cm^{-1} with the one at 1635 cm^{-1}, we can see that its signal is strengthened as the MMA content in the copolymer is increased. Moreover, we proceeded to a comparison of the spectra of the solid copolymers with a mixture of the liquid monomers containing 35%mol MMA (**Figure S1 b**). It is clear that any remaining unreacted monomers can be hardly detected in the spectra of the copolymers.

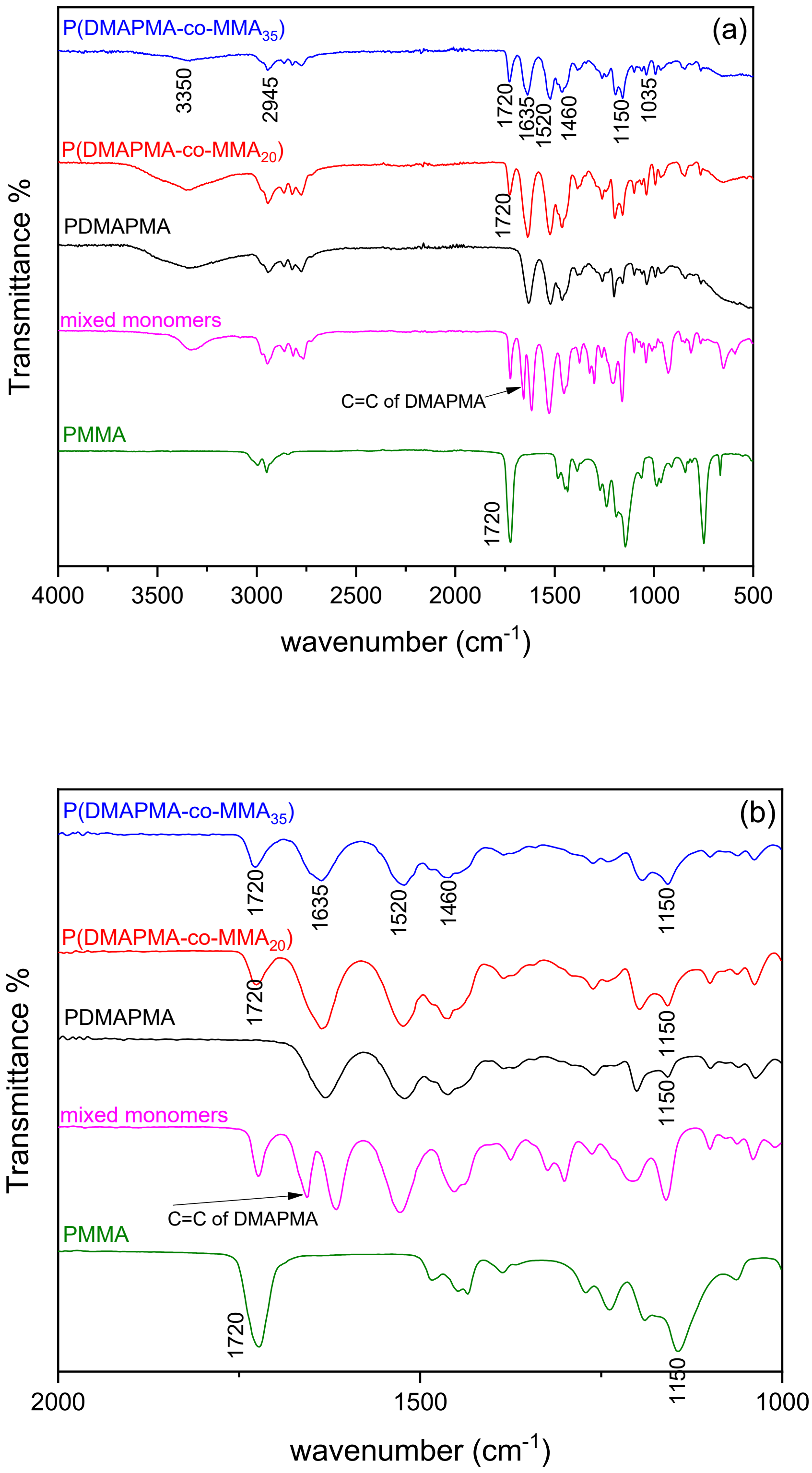

Figure S1. (a) ATR-FTIR spectra of PDMAPMA and PMMA homopolymers, the P(DMAPMA-co-MMA$_y$) copolymers and a mixture of the liquid monomers containing 35%mol MMA. (b) a magnification of the wavenumber area 1000 - 2000 cm^{-1} of Figure S1 a.

Table S1. pH values of the prepared buffer solutions and pH values of each solution after the addition of the copolymer P(DMAPMA-co-MMA_{35}) at a concentration of 1 % w/v.

initial pH	pH after addition of the polymer
6.91	8.73
7.21	9.38
7.75	9.94
7.65	9.66

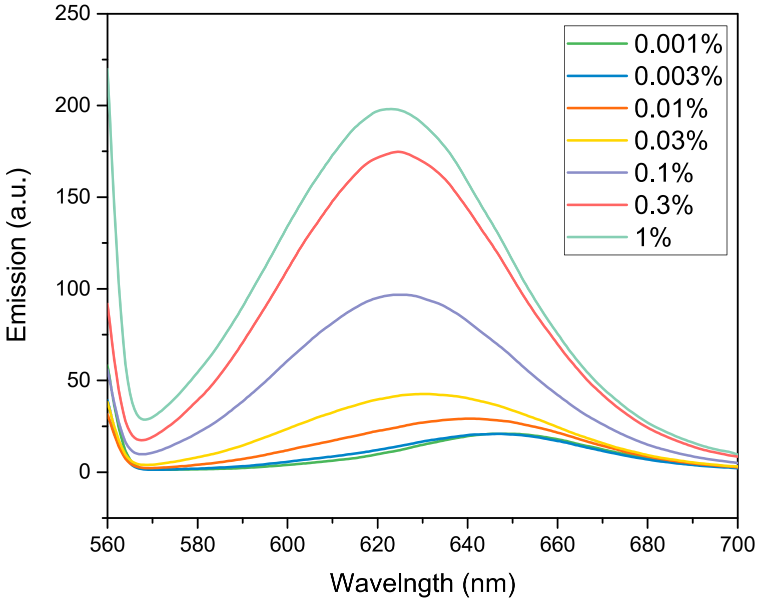

Figure S2. Emission spectra of Nile Red at different P(DMAPMA-co-MMA_{35}) polymer concentrations.

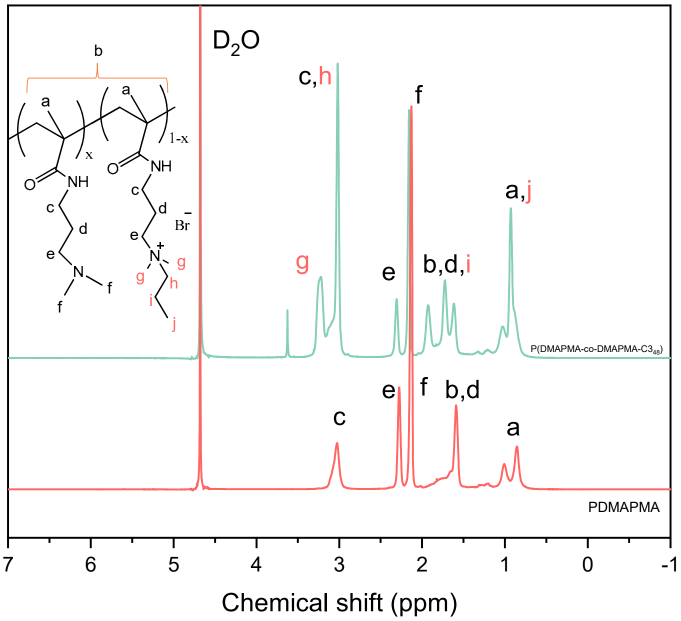

Figure S3. ^{1}H-NMR spectra in D_2O of the PDMAPMA homopolymer in combination with the 1-bromopropane alkylated polymer P(DMAPMA-co-DMAPMA-$C3_{48}$).

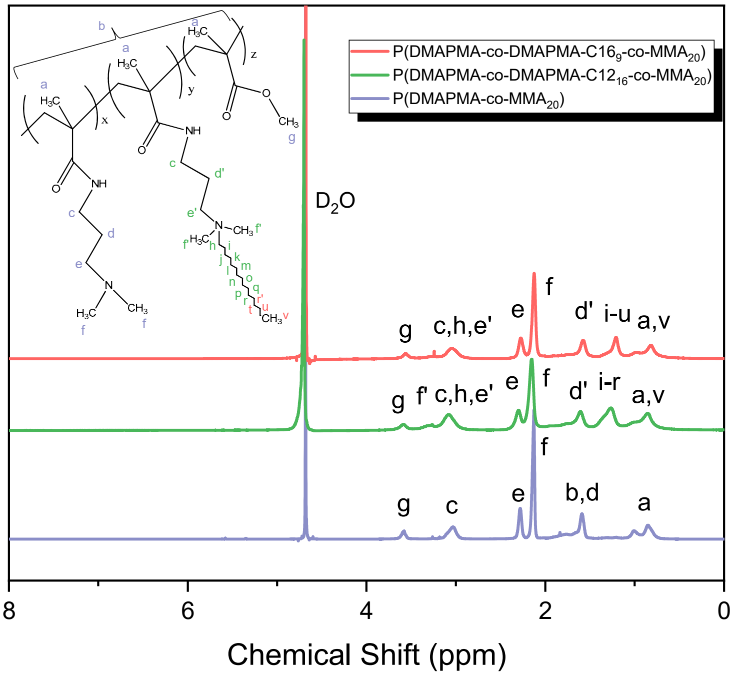

Figure S4. ^{1}H-NMR spectra in D_2O of the P(DMAPMA-co-MMA_{20}) copolymer (blue curve) in combination with the 1-bromododecane alkylated polymers (green curve) P(DMAPMA-co-DMAPMA-$C12_{16}$-co-MMA_{20}) and 1-bromodecahexane (red curve) P(DMAPMA-co-DMAPMA-$C16_9$-co-MMA_{20}).

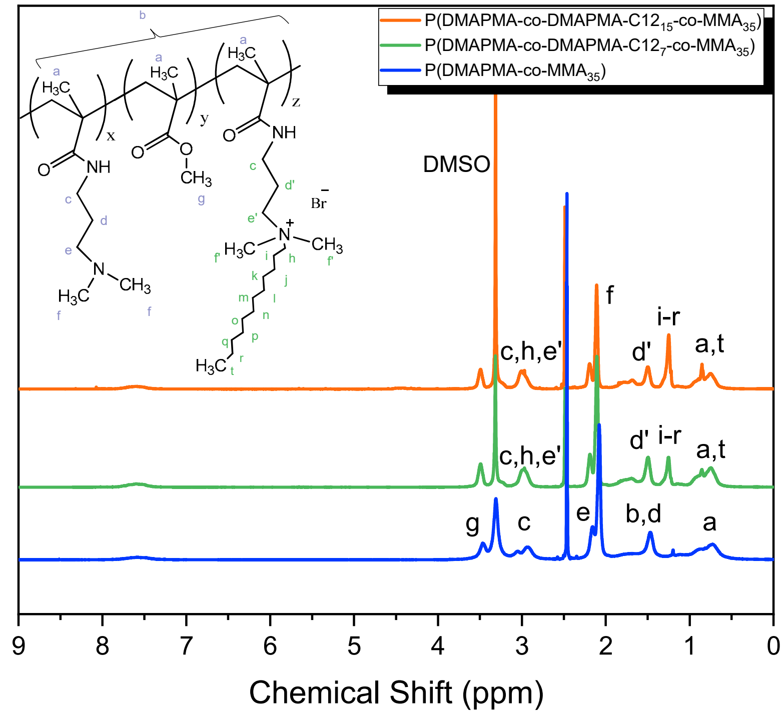

Figure S5. ^{1}H-NMR spectra in d6-DMSO of the copolymer P(DMAPMA-co-MMA$_{35}$) (blue curve) in combination with the alkylated polymers with 7 % 1-bromododecane (green curve) P(DMAPMA-co-DMAPMA-C12$_{7}$-co-MMA$_{35}$) and 15 % (orange curve) P(DMAPMA-co-DMAPMA-C12$_{15}$-co-MMA$_{35}$).

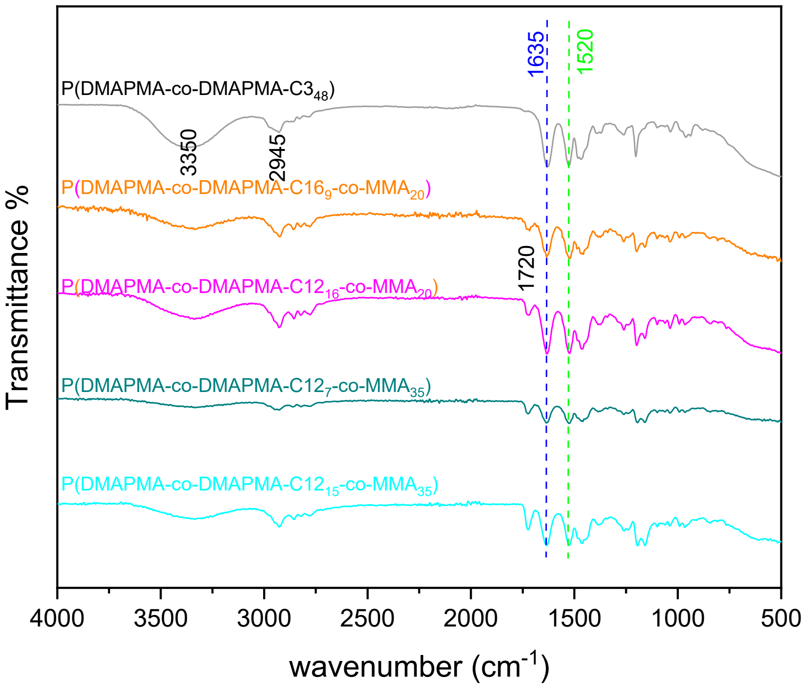

Figure S6. ATR-FTIR spectra of the alkylated polymers.

From the ATR-FTIR characterization (**Figure S6**), it is clear that the peaks at 3350, 1635 and 1520 cm^{-1} corresponding to N-H stretching and the amide I and amide II peaks, respectively, are still observed in the alkylated products. The presence of these peaks strongly supports that alkylation of tertiary amine groups takes majorly place. This is also supported by the fact that in Figure S5, the peak at ~7-8 ppm attributed to NH is observed in the ^1H NMR spectra of the alkylated products in organic d6-DMSO solvent.

The variation with temperature of the size distributions of P(DMAPMA-co-MMA_{35}) and alkylated P(DMAPMA-co-$DMAPMAC12_7$-co-MMA_{35}) copolymers is shown in **Figures S7** and **S8**. For P(DMAPMA-co-MMA_{35}), a major population with a size indicative of possible unimers is observed below 40°C, whereas a single population with a much higher size, indicative of larger aggregation, is observed above 40°C. In the case of P(DMAPMA-co-$DMAPMAC12_7$-co-MMA_{35}), a population with a size of 120-220 nm dominates, regardless of temperature.

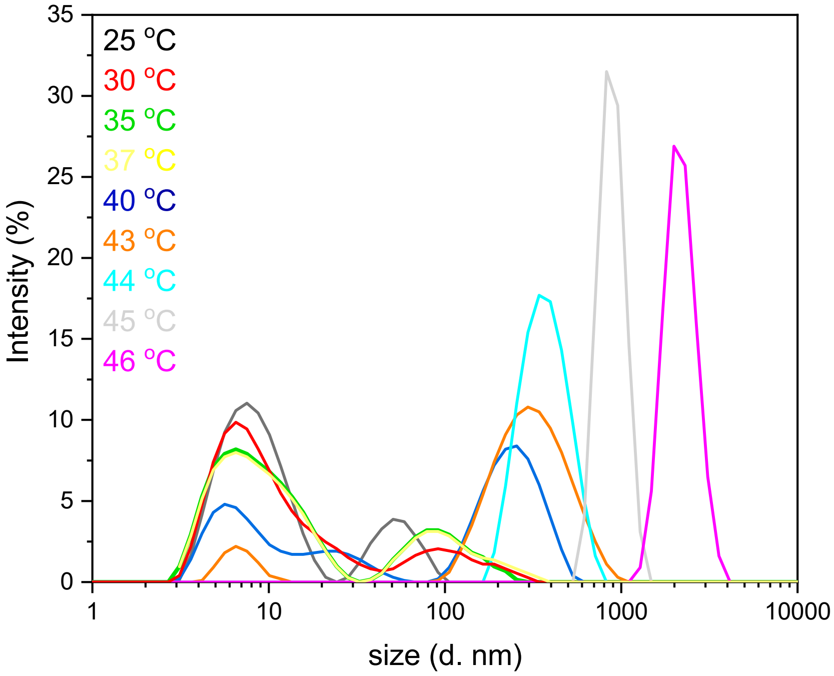

Figure S7. Intensity weighted particle size distribution of 1 % w/v non-alkylated P(DMAPMA-co-MMA_{35}) copolymer aqueous solutions obtained from DLS measurements, at different temperatures.

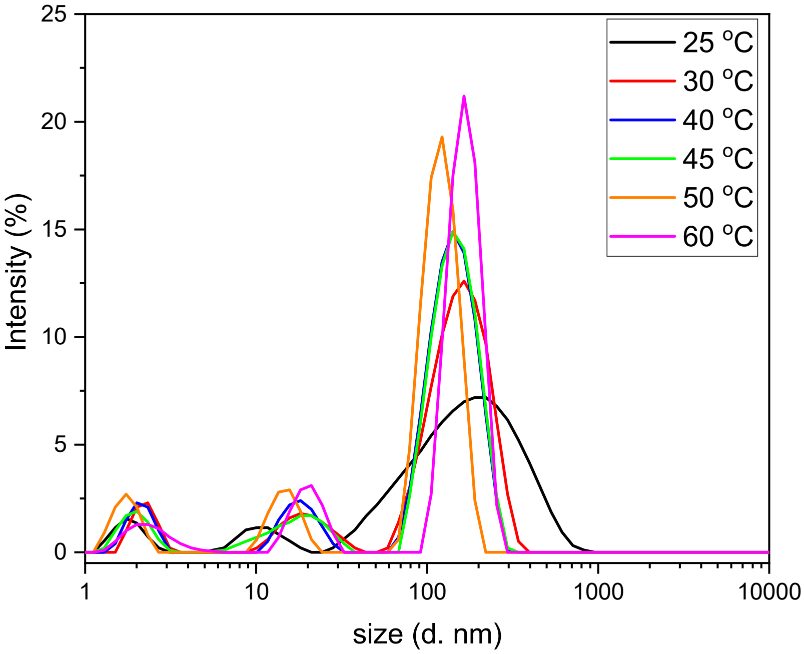

Figure S8. Intensity weighted particle size distribution of 1 % w/v alkylated P(DMAPMA-co-DMAPMAC12$_7$-co-MMA$_{35}$) copolymer aqueous solutions obtained from DLS measurements, at different temperatures.

---

1 Mishra, R.K.; Ray, A.R. Synthesis and Characterization of Poly{N-[3-(dimethylamino) propyl] methacrylamide-co-itaconic acid} Hydrogels for Drug Delivery. *J. Appl. Polym. Sci.* **2011**, *119*, 3199-3206.
